# Orthodontic post-adjustment pain control with acupuncture

**DOI:** 10.1590/2176-9451.19.4.100-106.oar

**Published:** 2014

**Authors:** Daniela de Cassia Faglioni Boleta-Ceranto, Ricardo Sampaio de Souza, Sandra Silverio-Lopes, Nathalie Canola Moura

**Affiliations:** 1 Full professor, Paranaense University (UNIPAR).; 2 Full professor, Department of Orthodontics, UNIPAR.; 3 Professor, Graduate program, Brazilian Institute of Therapy and Education (IBRATE).; 4 Specialist in Orthodontics, UNIPAR.

**Keywords:** Orthodontics, Acupuncture analgesia, Pain

## Abstract

**Objective:**

This study aimed to evaluate the analgesic efficacy of systemic acupuncture therapy on the pain caused after orthodontic adjustments.

**Methods:**

An initial sample of 30 orthodontic patients with fixed appliances monthly adjusted was selected; however, only 11 participants completed the study. For this reason, final sample comprised these patients' data only. Initially, average pain levels were assessed at different periods by means of an analogue visual scale (VAS) for three months without acupuncture. In the following three months, the volunteers were submitted to systemic acupuncture sessions on Hegu (LI4) and Jiache (St6) points,before orthodontic adjustments were carried out.

**Results:**

Results revealed statistically significant reduction in pain level indexes both for men (P = 0.030) and women (P = 0.028) when acupuncture therapy was performed prior to orthodontic adjustment. Patients did not present any side effects.

**Conclusion:**

Acupuncture is a safe and effective method in reducing orthodontic post-adjustment pain.

## Abstract (pt)

**Objetivo:**

o presente trabalho objetivou avaliar a eficácia analgésica da acupuntura sistêmica, realizada previamente, sob a dor oriunda pós-ajuste ortodôntico.

**Métodos:**

uma amostra de 30 indivíduos usuários de aparelhos fixos foi selecionada, os quais tinham de ser ajustados mensalmente. Contudo, somente 11 participantes completaram o estudo, e somente os seus dados foram coletados e analisados. A princípio, foi realizada uma média do índice de dor, em diferentes períodos, por meio de uma escala analógica visual (EAV), por três meses, sem a utilização da acupuntura. Nos três meses seguintes, os voluntários foram submetidos a sessões de acupuntura sistêmica nos pontos Hegu (IG4) e Jiache (E6), previamente ao ajuste ortodôntico. A média dos resultados do índice de dor com e sem acupuntura foram comparados.

**Resultados:**

os resultados demonstraram que houve redução estatisticamente significativa no índice geral de dor, tanto para os homens (p = 0,030) quanto para as mulheres (p = 0,028), após a utilização da acupuntura previamente ao ajuste, e nenhum voluntário apresentou efeitos adversos.

**Conclusão:**

conclui-se que os pontos de acupuntura utilizados são eficientes e seguros na redução da dor pós-ajuste ortodôntico.

## INTRODUCTION

Dental therapy in general, including orthodontic treatment, usually causes pain or distress. Pain is considered a very subjective symptom, for this reason, it is best defined as: "An uncomfortable sensory and emotional experience associated with real or potential injuries or described in terms of such injuries".^1^ Individual's past experiences, emotional state, cultural background, age and sex^2,3^ are among the facts that influence pain level, all of which may pose difficulties in measuring symptoms.

The literature shows that all orthodontic procedures, such as separator placement, fixed orthodontic appliances installation and activation, and orthopedic force application, cause pain. Additionally, fixed appliances tend to cause more pain than removable or functional ones, and the correlation between applied forces and pain is small.^4^

There are two major forms of distress caused by fixed orthodontic appliances: traumatic ulcers and pain during orthodontic movement.

In case of traumatic ulcers, orthodontic appliance functions as a traumatic factor that lesions the mucosa and causes epithelial tissue loss, thereby exposing subjacent conjunctive tissue and causing pain by local nociceptors stimulation.

As for orthodontic movement, there are no doubts that pain perception is part of the inflammatory reaction caused by blood flow alterations after orthodontic forces are applied. As a result, many chemical mediators are released as follows: Substance P, histamine, encephalin, dopamine, serotonin, glycine, glutamate, gamma-aminobutyric acid (GABA), prostaglandin, leucotriene, cytokine; thereby causing local hyperalgesia.^4,5^ Bergius et al^3^ analyzed 203 orthodontic patients, and demonstrated that 91% of them reported pain caused by orthodontic appliance, whereas 39% reported pain in every orthodontic appointment while adjustment was carried out. Studies show that nearly 95% of patients under orthodontic treatment present different levels of discomfort,^6,7^ especially within the first 24 hours after orthodontic adjustment. Pain caused by fixed orthodontic treatment gradually increases 4 hours after appliance adjustment, returning to normality on the seventh day.^2,7,8,10^

Studies reveal that the first 48 hours of pain after orthodontic adjustment cause as much disturbance as to interfere in patient's sleep and induce the need for medication. Krishnan^4^ conducted a literature review and found that nearly all orthodontic patients report moderate to extreme difficulty in chewing and swallowing solid food because of pain, proving that orthodontic pain can also interfere in patient's diet, thus raising another major concern for patients and professionals.

Erdinç and Dinçer^11^ reported that nearly 50% of their patients suffered pain within 6 hours to two days after orthodontic adjustment, which interfered in their daily activities. The authors also reported a reduction in pain intensity and the number of patients with pain from the third day on.

Considering the high rate of patients complaining about pain suffered during orthodontic treatment, different methods have been tested for its control, namely: low-level laser (LEDs) application, transcutaneous electrical nerve stimulation (TENS), neural stimulation, vibration to stimulate periodontal ligament, among others resulting in pain control.^5^

In addition to conventional techniques, researchers have sought new procedures for pain control. As a result, Dentistry is trying alternative methods to aid professionals bring more comfort to their patients. Nevertheless, new methods do not include the development of new ultra-modern equipments and/or last generation drugs, only. Researches show that ancient techniques are scientifically efficient in pain control, and acupuncture is among the most effective ones.

The present study assessed the analgesic efficacy of systemic acupuncture therapy on pain caused after orthodontic adjustments. The tested hypotheses were whether acupuncture was efficient or not in reducing pain caused by orthodontic adjustment.

## MATERIAL AND METHODS

This research was approved by the Ethics Committee on Human Research under protocol 00080375000-08. All volunteers filled an informed consent form.

This research was conducted as a blind study. The researcher responsible for acupuncture was aware of the treatment modality each patient would be submitted to. However, results were analyzed by a statistician unaware of the treatment to which each volunteer was subjected to.

Patients under fixed orthodontic treatment at the Graduate Program Orthodontic Clinic participated in this research. First, research volunteers were selected. To be included in the group, the patient had to be complaining of pain after orthodontic adjustment and accept acupuncture treatment. Thirty patients volunteered, although, only 11 (7 women and 4 man) concluded the study. Patients' mean age was of 16.2 years (Table 1). The volunteers were monthly assessed during 6 months by means of a visual analogue scale (VAS) for pain. Each scale corresponds to a 10-cm horizontal line with two points meaning "no pain" and "the worst pain possible". Participants were instructed to mark a transversal trace on the line, representing the equivalence of pain intensity they felt.^3^

**Table 1:** Volunteers' age and sex.

Sex	Mean ± SD
Female	15.33 ± 3.933
Male	17.4 ± 3.43
Total	16.2 ± 3.68

VAS allowed pain to be quantified during different periods (before orthodontic adjustment, right after adjustment, 4, 8, 24 and 72 hours after adjustment). Each participant received 7 scales every month in accordance with previously described periods. In the following appointments, the scales were filled and collected for analysis which measured each scale with a millimetric ruler so as to obtain numerical data.^3^

Within the first 3 months, the volunteers were instructed to fill out VAS after orthodontic adjustment so as to obtain an average of participant's pain without acupuncture treatment. In the following 3 months, antisepsis of the areas of needle insertion was done with cotton and 70% alcohol 5 minutes prior to each orthodontic adjustment. Subsequently, systemic needles were inserted at Hegu (LI4) and Jiache (St6) points on both sides. The needles remained in place for twenty minutes.^12^ Dragon^®^ Sterile stainless steel needles (0.20 x 25 mm) were used.

New VASs were filled out during the same periods previously described. Results were used to calculate the average pain described by each patient during the three orthodontic adjustment appointments after acupuncture therapy. Therefore, volunteers performed self-analyses.

Patients were instructed not to take any other analgesic medication while the effect of acupuncture therapy was being analyzed. Should pain be too intense so as to require analgesic medication, the instruction was to record the date, time, type and doses of medication.

Analysis of results of the 11 volunteers was carried out with Sigma-Stat statistic program. Data was first submitted to analysis of variance (ANOVA) (P ≤ 0.05). Should ANOVA yield significant values, Tukey test was used to identify significant differences between the average pain level obtained through VAS.

## RESULTS

Since significant statistic differences were found in pain perception between male and female patients, results were separately assessed according to that variable.

### Variation of pain perception according to time

**Figure 1:**
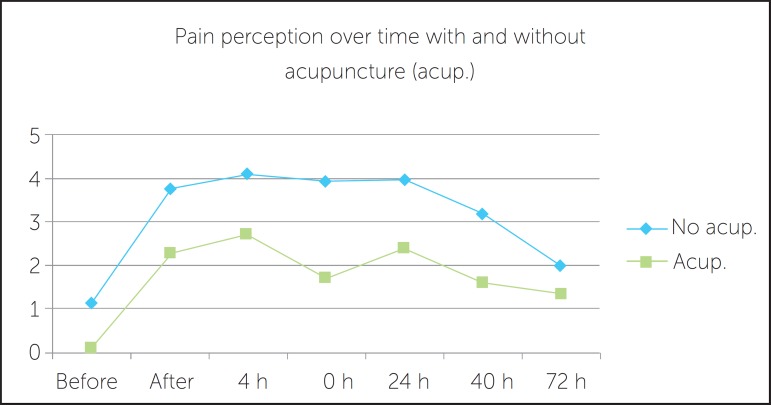
Average pain perception alteration over time, with and without previous acupuncture therapy.

Results reveal a gradual increase in volunteers' pain level right after orthodontic adjustments and within the first 24 hours with gradual reduction during the following periods. Table 2 and Figure 1 show the averages between female and male patients.

**Table 2:** Average pain level alterations over time.

	Before*	After**	4 h	8 h	24 h	48 h	72 h
Without acupuncture	1.08	3.76	4.09	3.96	3.965	3.2	2
With acupuncture	0.104	2.2504	2.711	1.71	2.4	1.6	1.29

* Before orthodontic adjustment

** Right after orthodontic adjustment.

### General pain level average of male and female volunteers with and without acupuncture therapy

**Figure 2:**
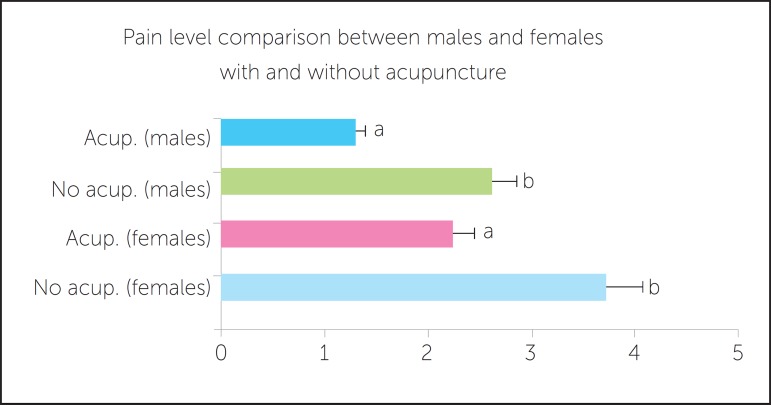
Average (and standard deviation) of male and female volunteers' pain level after orthodontic adjustment with or without previous acupuncture therapy. The comparison using distinct letters through Tukey test statistically distinguishes the averages among themselves

Analysis of variance (ANOVA) followed by Tukey test at a 5% significance level was conducted between male and female patients and showed a statistically significant difference in pain level after orthodontic activation with or without previous acupuncture therapy. Results are shown in Table 3 and Figure 2.

**Table 3:** Average values of pain level after orthodontic adjustment with or without previous acupuncture therapy in male and female volunteers. Data expressed as mean ± standard deviation.

Treatment	Mean ± SD
Without acupuncture male	2.61 ± 1.263
With acupuncture male	1.27 ± 0.677
Without acupuncture female	3.72 ± 1.129
With acupuncture female	2.22 ± 1.113

### Average pain level of female volunteers during different periods with and without acupuncture

**Figure 3:**
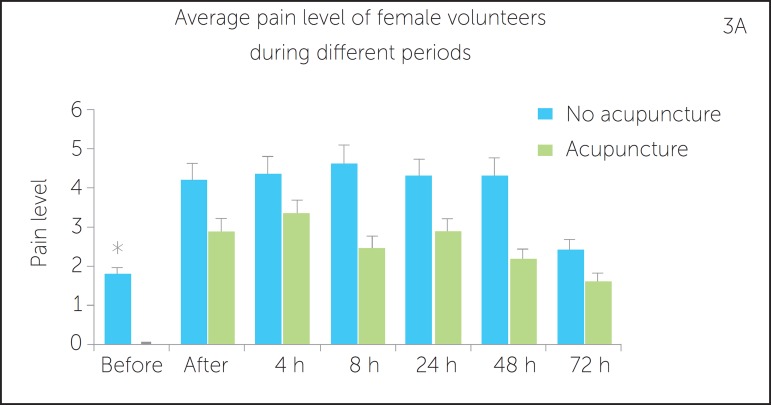
Average pain level (and standard deviation) of female (**A**) and male (**B**) volunteers during different periods of orthodontic adjustment with and without previous acupuncture treatment. * p < 0.05.

Averages obtained with or without acupuncture treatment at different studied periods submitted to analysis of variance (ANOVA) at 5% significance level between male and female patients showed that although there was a reduction in pain level during all periods when acupuncture was performed before orthodontic adjustments, it was only statistically significant during the pre-orthodontic adjustment period. Results are shown in Table 4 and Figure 3A.

**Table 4:** Average values of pain levels in different periods with or without previous acupuncture (acup.) treatment in female and male volunteers. Data expressed as mean ± standard deviation.

	Before	After	4 h	8 h	24 h	48 h	72 h
No acup. (female)	1.77 ± 1.68	4.21 ± 2.69	4.36 ± 2.39	4.63 ± 1.84	4.3 ± 2.22	4.31 ± 1.88	2.43 ± 1.90
Acup. (female)	*0.02 ± 0.03	2.92 ± 2.34	3.34 ± 2.11	2.50 ± 2.49	2.90 ± 2.64	2.20 ± 1.94	1.64 ± 2.03
No acup. (male)	0.4 ± 0.62	3.32 ± 1.77	3.83 ± 1.67	3.3 ± 1.64	3.63 ± 1.96	2.22 ± 1.52	1.6 ± 2.38
Acup. (male)	0.18 ± 0.29	1.59 ± 2.10	2.08 ± 2.17	*0.93 ± 0.39	2.03 ± 0.88	1.18 ± 2.39	0.95 ± 1.54

* p < 0.05.

### Average pain level of male volunteers during different periods with and without acupuncture

Analysis of variance (ANOVA) was performed at 5% significance level among male participants during different periods with and without acupuncture and revealed a statistically significant difference in pain level 8 hours after orthodontic adjustment, only. Nevertheless, there was a visual reduction in the levels of pain perception recorded during all periods. Results are shown in Table 4 and Figure 3B.

## DISCUSSION

Even if analgesic and anesthetic medications are used, dental therapy, including orthodontic treatment, is acknowledged by most people to cause pain or even a light distress. The literature shows that all orthodontic procedures, such as separator placement, bracket installation and activation, and the application and activation of orthopedic forces, cause pain. Additionally, fixed appliances cause more pain than removable or functional ones and the correlation among applied forces and pain felt by patients is small.^4^

Among the facts that influence pain level are: former experiences, emotional state, cultural background, age and sex,^2,3,18^ all of which may pose difficulties in measuring the symptom. Except for patient's age and sex, which can easily be assessed, all other factors are quite personal and can be considered as limitations of the present study, since they were not considered as variants that influence pain levels and could interfere in the results.^3^ Participants' motivation and expectations towards the acupuncture analgesic effect also seem not to influence the results. Since the average values were monthly collected, participants had no access to former analyses and could hardly compare the records in a way that allowed them to forge final results. This could be considered a limitation of this study. Ideally, Sham acupuncture (needle applications at non-analgesic points) should have also been used; however, once a great number of participants quit, it was impossible to divide the group due to the limited number of volunteers comprising the sample. Further studies will be able to correct this fact.

Considering the high rate of patients complaining about pain suffered during orthodontic treatment, different methods have been tested for its control, namely: Low-power laser (LEDs) application, transcutaneous electrical nerve stimulation (TENS), neural stimulation, vibration to stimulate periodontal ligament, among others resulting in pain control.^5^ In addition to conventional techniques to control pain, Dentistry has introduced alternative methods to aid professionals bring more comfort to their patients. Those alternative techniques have been acknowledged and approved by the Federal Council of Dentistry (Resolution 82, July, 2008), and can be applied to reduce pain in dental patients. Acupuncture is one of the most effective techniques.

The aim of the present study was to assess the analgesic efficiency of systemic acupuncture performed before orthodontic adjustment to treat orthodontic post-adjustment pain. The research started with 30 patients gathered from an Orthodontic Graduate Program, but only 11 participants concluded it. Some degree of desistance is expected during human research. No volunteers declined to participate in this study due to its methodology. A great number of absences during orthodontic adjustment appointments were observed, thereby eliminating some participants. In some cases, the orthodontist responsible for the orthodontic adjustment was absent, which also hindered patient's particiation on the research. All volunteers had their pain level analyzed by Visual Analogical Scales (VAS).

VAS was chosen due to being greatly used to measure pain and having some advantages among verbal scales, such as the facility children have in filling it out.^13^ Patients were asked to register the intensity of pain they felt on the line representing the estimate variable.^13^ A numerical value was assigned by the researcher using a millimetric ruler. Consequently, precise values were not possible.^14^ No volunteers had difficulties in filling out the scales, reinforcing their easiness.

Bergius et al^3^ assert that pain caused by orthodontic adjustment can be immediate or delayed. Immediate pain occurs after the archwire is attached and periodontal ligament is compressed. The latter starts a few hours later when hyperalgesia of periodontal ligament generates allodynia through the release of chemical active substances (as prostaglandin, histamine and substance P), thereby causing partial depolarization of afferent fibers, neural facilitation and peripheral sensitization. When an orthodontic force is applied to a tooth, an initial distress and/or pain phase of 1 or two days usually occur.^10^ The intensity of pain generally increases within 4 to 24 hours and then reduces to normal levels within a 7-day period.^3^ There is generally a circadian variation in pain intensity, with an increase of pain between late afternoon and night, although that does not intensely interfere in sleep.^8^ Our results confirm the fact that pain begins after orthodontic adjustment, its apex occurs within 8 to 24 hours and gradually reduces over time, even with previous acupuncture treatment. However, the present work shows that intense decrease occurs when acupuncture treatment is previously performed.

Scheurer^7^ also showed that general pain intensity perception, analgesic taking, pain during feeding and distress influence on daily life were significantly greater in women than men. Women are more sensitive to experimental painful stimuli than men, with clinical pain of greater frequency and severity lasting for a longer period of time and in a larger range of body locations.^15,16^ In the present study, we also observed greater feminine pain sensitiveness, with or without previous acupuncture treatment. This fact could be related to women's great hormonal variability, mainly in female teenagers voluntaries. For both males and females, acupuncture treatment significantly reduced pain after orthodontic adjustments, thus proving its efficiency.

Even when results showed a reduction in pain levels after acupuncture treatment during all studied periods (both for men and women), it was only statistically significant during two periods. Before orthodontic adjustment in female patients, which is justified by their hormonal alterations - a limitation for the study since their menstrual period was not assessed. For men, within 8 hours after adjustment, corresponding, according to the literature, to the time of pain apex.^3^

Pain reduction observed in the present study probably occurred due to acupuncture action mechanism, activating descending pain inhibition and antinociceptive substances release, such as β-endorphin (analgesics), cortisol (anti-inflammatory) and serotonin (antidepressant) in the blood flow and cephalorachidian liquid.^17^

Data obtained in the present study confirm the efficiency of acupuncture as a complementary treatment in Orthodontics. It is important since nearly all orthodontic patients, as described in many studies, report moderate to extreme difficulty in chewing and swallowing solid food because of pain, proving that orthodontic pain can also interfere on patient's diet, a major concern for patients and professionals.^4^ Also, Erdinç and Dinçer^11^ reported that nearly 50% of their patients suffered from pain that interfered on their daily activities within 6 hours to two days after orthodontic adjustments, which could be diminished by stimulation of analgesic points through acupuncture, as shown in the present study.

The literature highlights many analgesic acupoints. Microsystems of acupuncture such as ear acupuncture, hand acupuncture and scalp acupuncture points are another treatment option, instead of systemic acupuncture. Hegu (LI4) and Jiache (St6) points were used for its location and professional easy access.

Acknowledgment and approval of acupuncture practice as a complement to dental treatment, issued by the Federal Council of Dentistry in July 2008, will certainly bring progress in this matter, since national researches using acupuncture for dental treatment are few and, for this reason, narrow the benefits of the method for both patients and professionals. Acupuncture does not produce side effects and can be safely practiced by a qualified professional. In this study, a total of 33 acupuncture sessions were done (3 on each one of the 11 volunteers), and no patients presented side effects related to needle insertion.

The fact that it is not an onerous technique is important and must also be observed, particularly considering the present social-economic situation in Brazil.

## CONCLUSION

Within the limitations and conditions of this experiment, it is reasonable to conclude that systemic acupuncture treatment performed before orthodontic therapy can reduce pain level in both men and women. Additionally, acupuncture proves to be a safe technique employed for this purpose.
